# A Novel Infrared and Visible Image Fusion Approach Based on Adversarial Neural Network

**DOI:** 10.3390/s22010304

**Published:** 2021-12-31

**Authors:** Xianglong Chen, Haipeng Wang, Yaohui Liang, Ying Meng, Shifeng Wang

**Affiliations:** 1 School of Optoelectronic Engineering, Changchun University of Science and Technology, Changchun 130022, China; 2018001244@mails.cust.edu.cn (X.C.); wanghaipeng@mails.cust.edu.cn (H.W.); 2018001297@mails.cust.edu.cn (Y.L.); sf.Wang@cust.edu.cn (S.W.); 2 Key Laboratory of Optoelectronic Measurement, Optical Information Transmission Technology of Ministry of Education, School of Optoelectronic Engineering, Changchun University of Science and Technology, Changchun 130022, China; 3 Zhongshan Institute, Changchun University of Science and Technology, Changchun 130022, China

**Keywords:** image fusion, adversarial neural network, face recognition, infrared images, visible images

## Abstract

The presence of fake pictures affects the reliability of visible face images under specific circumstances. This paper presents a novel adversarial neural network designed named as the FTSGAN for infrared and visible image fusion and we utilize FTSGAN model to fuse the face image features of infrared and visible image to improve the effect of face recognition. In FTSGAN model design, the Frobenius norm (*F*), total variation norm (*TV*), and structural similarity index measure (*SSIM*) are employed. The F and TV are used to limit the gray level and the gradient of the image, while the *SSIM* is used to limit the image structure. The FTSGAN fuses infrared and visible face images that contains bio-information for heterogeneous face recognition tasks. Experiments based on the FTSGAN using hundreds of face images demonstrate its excellent performance. The principal component analysis (PCA) and linear discrimination analysis (LDA) are involved in face recognition. The face recognition performance after fusion improved by 1.9% compared to that before fusion, and the final face recognition rate was 94.4%. This proposed method has better quality, faster rate, and is more robust than the methods that only use visible images for face recognition.

## 1. Introduction

Face recognition is one of the main applications of machine vision and plays a significant role in data security field. Currently, most of the face information still uses visible imaging systems to capture face images and relies on feature extraction algorithms [1]. The research on face recognition in a controlled environment has proved to be successful and achieved wide application. There are still major challenges for an uncontrolled environment, where the subjects are dynamic, and it is difficult to capture changes in the machine angle due to several reasons [1]. Face information is prone to being influenced and partially reduced in the dark environment. The influential factors such as the hairstyle and clothing can also affect the face bio-information. The complex environment poses a serious challenge for both the security surveillance and daily application. In a society with extremely developed machine vision, the technology of using digital tools to forge face data already exists, such as neural network face reconstruction and 3D face model attack. The data in the visible band alone cannot provide reliable face digital information.

We urgently need more advanced technology to improve the characteristics of a face. The accuracy of face recognition should be further improved to prevent the attack of a virtually forged face image [1]. Therefore, researchers have designed different sensing devices to capture the face information more precisely. However, it is unrealistic to use many expensive machines for face recognition. Therefore, a breakthrough in software is needed to find a more suitable, safer and faster face recognition algorithm.

Currently, traditional algorithms are applied to image fusion, such as HSV transform fusion [2], IHS transform fusion [3], and filter fusion [4]. Researchers have started to explore image fusion applications with the increase in popularity of deep learning. Deep learning exhibits extremely high image fusion capability and obviously surpasses several traditional image fusion algorithms. Besides, it provides excellent exploring conditions for image fusion. For example, when transforming an image by the HSV transform fusion [2], the pixels of the image are rounded and inverted after transforming the high-frequency domain with the low-frequency domain, which leads to an irreversible image loss in the H, S, and V domains. Due to this loss, the local information will be blurred.

However, in the field of deep learning, generative adversarial networks (GANs) [5] have achieved remarkable results when being applied to image fusion. The GANs proposes a new network to estimate generative models by an adversarial process. In this process, two models are trained simultaneously: a generative model *G* for estimating the data distribution and a discriminative model *D* to predict the probability that a sample is from the training set, i.e., the real dataset, and not *G*. For *G*, the training process maximizes the probability that *D* is incorrect in its judgments. The GAN’s original objective function is:

(1) $$\underset{G}{\min}\;\underset{D}{\max}V\left(D,G\right)=E_{x∼p_{\mathrm{data}\left(x\right)}}\left[\log D\left(x\right)\right]+E_{x∼p_{z\left(x\right)}}\left[\log\left(1-D\left(G\left(Z\right)\right)\right)\right]$$

where *G* represents the generator, *D* represents the discriminator, x represents the real data, $p_{data}$ represents the real data probability density distribution, and *Z* represents the random input data, which is random Gaussian noise.

The above equation shows that the discriminator wants to distinguish as much as possible between the true sample x and the false sample G(Z). Therefore, D(x) must be as large as possible and D(G(Z)) as small as possible, and V(D,G) should be as large as possible. The generator *G* wants its generated false data G(Z) to fool the discriminator *D* as much as possible. It wants D(G(Z)) to be as large as possible and V(D,G) to be as small as possible. The two modules of the GAN train against each other and finally reach the global optimum. It uses the visible image as the discriminator, and the mixed band superimposed images as the generator. During the model training, the generator can migrate image features, which compensates for the partial loss of local image information caused by fusion. Thus, the GAN would obtain a more desirable image fusion performance than the traditional algorithm. Nevertheless, there are a few limitations of deep learning compared with the traditional algorithms. Deep learning is exceptionally dependent on the graphics card performance in image processing, while the traditional algorithms can produce results just using the CPU. The traditional algorithm is better for image fusion speed, while deep learning is better in terms of the image fusion accuracy. In this paper, an image fusion method named the FTSGAN was proposed, which can be executed using the graphics card and can fuse images more rapidly than using the CPU.

**Figure 1:**
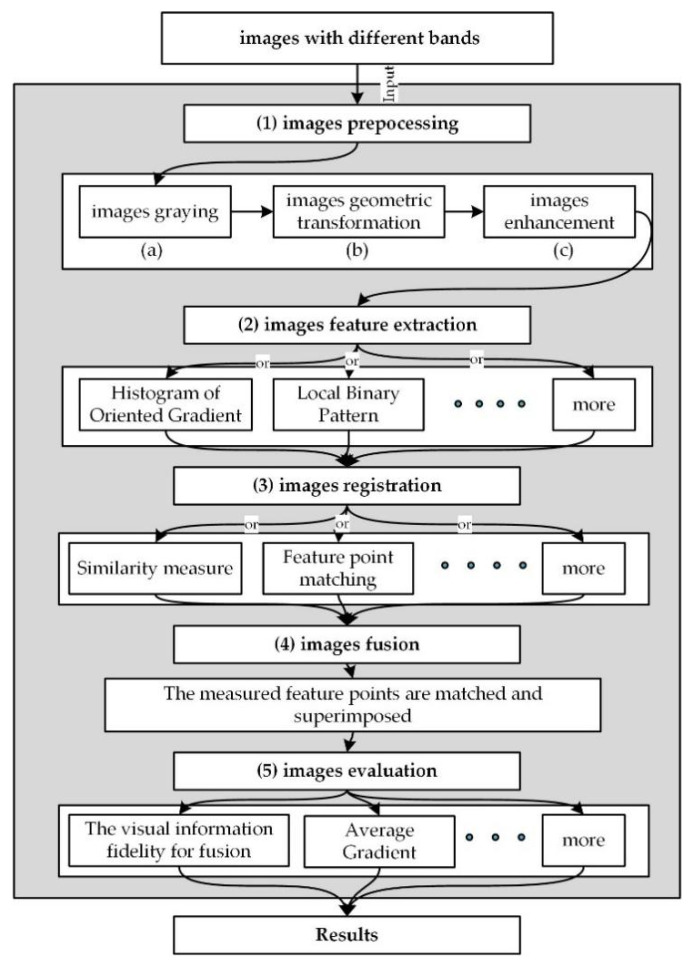
General images fusion process. First, the grayscale processing is to avoid band distortion. Second, features of the image are extracted as the feature points of the image. Third, the image can be matched at the corresponding feature points, and then a variety of algorithms are used to complete the image fusion. Finally, we evaluate the fused image to see if the ideal image fusion effect is completed.

This paper aims to use the original GANs [5] structure as a foundation to further rebuild its superstructure. The face bio-information was fused using the constructed new model with the powerful feature migration capability of the GAN model itself. With the increasing demand for image features, the feature extraction is increasingly relying on the image fusion using the mathematical method. Figure 1 shows the basic image fusion procedure, which consists of image preprocessing, image feature extraction, image alignment, image fusion and image evaluation processes. All these processes were implemented using different algorithms.

As image fusion research originated in the early 1970s, the technology was applied immediately to the military field. The US research institutions located the enemy ships accurately by fusing multiple independent sonar signals. In the 1980s, the researchers started to focus on image fusion and made remarkable achievements. Since then, the image fusion techniques began to play a significant role for civilian use [6]. The transform domain fusion method is an image fusion method and includes the IHS transform [3], PCA transform [7], and HPF transform [8]. There are spatial region fusion methods, such as the simple combinatorial image fusion method [9] and logical filter fusion [10]. Since the 1980s, no unified evaluation or unified image fusion models have been proposed, and thus, it is urgent to design different image fusion algorithms.

Along with the emergence of deep learning [11], researchers have made breakthroughs in image fusion algorithms. The GANs have a great potential as a mathematical model for image fusion. The most typical examples include the DDcGAN [12] and FusionGAN [13] models. The models further enhance the image fusion in infrared and visible bands, allowing for more explicit features and faster fusion. In this paper, we designed a new adversarial neural network model for image fusion. Since we used F, TV, and SSIM, this new model was named as the FTSGAN.

## 2. Methods

### 2.1. Design of Image Fusion Method

#### 2.1.1. Image Fusion Process Design

**Figure 2:**
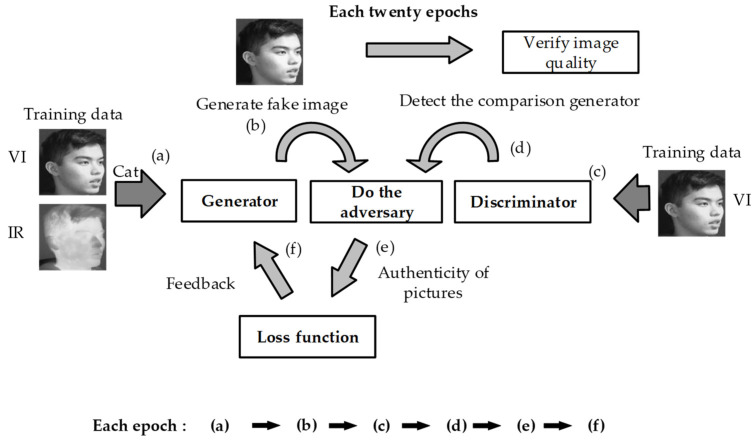
The FTSGAN model training process. (‘Cat’ is an application programming interface (API) for the neural network tool Pytorch and it is used to concatenate the matrixes.) We put the images into the generator and discriminator respectively for training. The images generated by the generator will compete with the discriminator to evaluate the effect of the generator, and then the obtained effect will be constrained by the loss function. It is necessary to repeat this state again and again that a good image generation effect is obtained.

The FTSGAN was designed to mitigate the GAN’s defects. It adopts the AutoEncoder neural network as the generator. The AutoEncoder neural network [14] consists of two parts—encoder and decoder—which can extract the features of the image more efficiently than the original GAN [5] structure. In Figure 2, the visible and infrared images are used as input, and subsequently the information is superimposed using concatenate. The superimposed data are input into the FTSGAN model. In this process, the visible face image works as the input of the discriminator and trains the generator for adversariality. Furthermore, the generator can produce adverse effects that can deceive the discriminator. It generates the fusion image effectively to integrate the infrared face information without losing the original image information.

#### 2.1.2. Design of Image Fusion Loss Function

The observation of the infrared dataset shows that the edge information of the infrared images is blurred, and the magnitude information is complicated. There is also existing information on interference gradient. The gradient information of visible images applied for fusion would have a gradient loss as the surrounding environment’s gradient decreases, which can blur the image. Thus, this fusion would make the image more unobservable as well as makes the blurred infrared image information appear as the noise signal.

The loss functions were designed to enable the visible image’s structure information to be preserved in the gradient. We divided the loss function of the generator, shown as follows, into three parts: $L_{G}^{a}$, $L_{G}^{b}$, and $L_{G}^{c}$:

(2) $$L_{G}=L_{G}^{a}+\left(1-L_{G}^{b}\right)+L_{G}^{c}$$

The $L_{G}^{a}\;\mathrm{is}\;\mathrm{given}\;\mathrm{as}\;E_{x∼p_{z\left(x\right)}}\left[\log\left(1-D\left(G\left(Z\right)\right)\right)\right]$, which is the negative result from the generator against the discriminator and part of Equation (1). We used the staking of the visible image and the infrared image instead of random noise *Z* in it. The *SSIM* [15] supervises the generator. The structure of the fused image converges more closely to the gradient of the visible image. As we wanted to keep more details in the visible component, we assigned the coefficients of 1 and 0.7 to the visible and infrared images, respectively. The loss function of the *SSIM* is as follows:

(3) $$L_{G}^{b}=E||1.7*G\left(v,i\right)-1*v-0.7*i|{|}_{SSIM}$$

For the intensity fusion, the *F* [16] and *TV* [17] also supervise the generator. The former can record the absolute value of the corresponding matrix function and will determine the difference of each image’s intensity. The intensity information is utilized in regulating and constraining fusion performance of the fused image. The coefficients are set to 0.7 and 0.5 for the visible and infrared images, respectively, in order to satisfy the human eye. The *TV* function reduces noise by means of variable differential equations. It facilitates the identification of the image gradient and makes the decomposition of the image clearer. We use it for constraining the visible light information of the generated image in order to generate better texture of the visible image. Thus, the coefficient of the *TV* function is given as 0.7. The loss function of the *F* and *TV* is given as:

(4) $$L_{G}^{c}=0.5*\left|\left|G\left(v,i\right)-i\right|{|}_{F}+0.7*\right|\left|G\left(v,i\right)-v\right|{|}_{F}+0.7*|\left|G\left(v,i\right)-v\right|{|}_{TV}$$

For the discriminator, we use GAN’s original loss function. The discriminator’s gross loss function is as follows:

(5) $$L_{D}=E\left|\left|-\log D_{v}\left(v\right)\left|\left|+E\right|\right|-\log\left(1-D_{v}\left(G\left(v,i\right)\right)\right)\right|\right|$$

#### 2.1.3. The FTSGAN Network Structure Design

**Figure 3:**
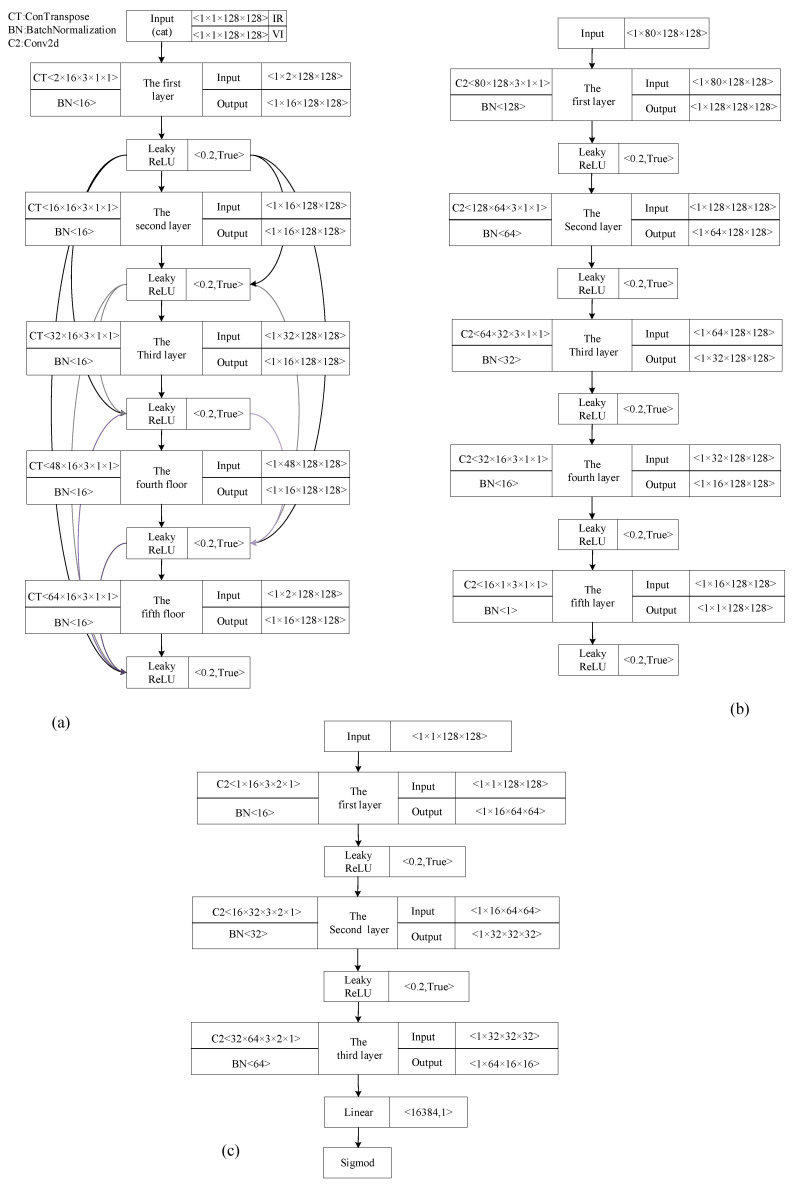
Structure of the FTSGAN: (**a**) Encoder structure, (**b**) decoder structure, (**c**) discriminator structure.

As Figure 3a,b show, the FTSGAN’s structure takes advantage of the GAN structure. It further promotes the generator’s [18] AutoEncoder neural network [14] to enhance feature extraction by simultaneously using the deconvolutional neural network [19] structure introduced in the encoder of the GAN [5] and the dense neural network [20] structure. The deconvolution structure can improve the image quality at low pixels. The dense neural network structure reduces the gradient loss and passes the gradient information from one layer to the next layer. As Figure 3a shows, we designed five layers of the deconvolutional neural network. For improved image quality, the dense neural network layer connection was used to connect the five deconvolutional layers. Furthermore, the LeakyReLU function was utilized in the encoder layer.

The encoder structure used a 3 × 3 kernel as the deconvolution kernel, with the sliding step and padding set to “1”. Starting from the output data of dimensions 1 × 2 × 128 × 128 from the first layer of the neural network, the input data of each layer will be concatenated by the input and output of the previous layer and used as input the next neural network. In addition, as the adopted image size was too small, the deconvolutional neural network improved the image features’ sampling quality. We apply the five convolutional neural network layers as the structure of decoder to ensure strong fitting ability. The LeakyReLU function is utilized to connect the five convolutional neural network layers, which makes the neural network converge and prevents the gradient from disappearing, as shown in Figure 3b.

As Figure 3c shows, the discriminator of FTSGAN applies three convolutional layers as the backbone and finally applies a linear layer as the output layer. The discriminator uses the visible images as input. Subsequently, the data generated by the generator are utilized against the discriminator. For the neural network structure, all layers can be normalized by means of a BatchNormalization structure. The LeakyRelu activation function will be used in each layer of the neural network structure to improve the sparsity, reduce the interdependence of parameters, and alleviate the over fitting problem. Its negative slope is set “0.2”. The speed of the neural network learning is significantly reduced at the same time.

### 2.2. Design of Face Recognition Method

**Figure 4:**
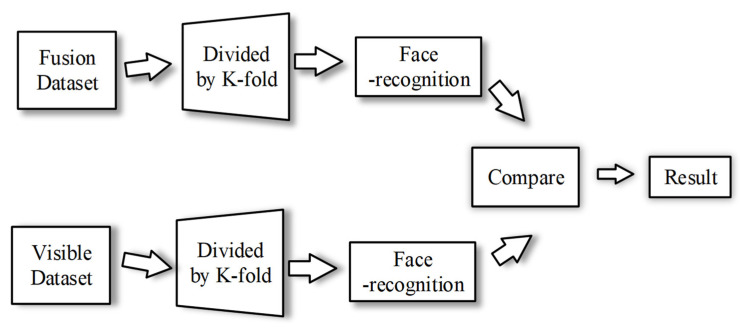
Overview of face recognition verification.

Figure 4 shows cross-validation of face recognition. We compare the recognition accuracies of visible and fused images by means of K-Fold cross-validation [21,22] to illustrate the advantages of the FTSGAN.

The principal component analysis (PCA) [23,24] which is an unsupervised dimensionality reduction method was applied to reduce face image dimensionality and obtain a low-dimensional face feature space. The linear discrimination analysis (LDA) [25,26] which is a supervised dimensionality reduction method with labels was applied to gain the fused face feature space on this low-dimensional face feature space. As Figure 4 shows, the PCA-LDA face recognition algorithm [27,28] was applied to carry out a face recognition experiment. The PCA algorithm, which downscales the high-dimensional space to the low-dimensional space, eliminated the singularity problem caused by the small sample size problems. Afterwards, the LDA algorithm for feature extraction aims to get the fused feature space and then project the training and test images to the fused feature space. The nearest neighbor classification was applied to determine the class to which the test image belongs. The PCA-LDA algorithm’s robustness was ensured using the six-fold cross-validation method to effectively decrease the error caused by the input sequence and the number of training samples. The number of correct classifications was divided by the total number of test images to obtain the face recognition accuracy.

## 3. Experiments and Discussion

### 3.1. Preparation of Experiments

Table 1 and Table 2 show the hardware configuration and the dataset used in the experiments, respectively. The IR2RGB dataset from Tufts University [29] was used. We download the IR2RGB dataset from Tufts University. As images of the other 73 sets partly are missing, we selected the 40 most accurate sets of face images from the 113 sets of face images and took an equal number of images into consideration in every set. There were 20 sets where each set consisted of face images with eyeglasses. In the remaining sets, each set consisted of face images without eyeglasses. Each set contained 12 different facial poses and facial expressions. Considering our limited picture dataset, the twelve face images were divided into six folds, and each fold had two face images.

### 3.2. Experimental Procedure

According to the design of image fusion algorithm, the following experimental procedure is designed and recorded in Table 3.

### 3.3. FTSGAN Training Process

Through the design of FTSGan model structure, we will train the model according to Table 4.

### 3.4. Face Recognition Authentication Process

**Figure 5:**
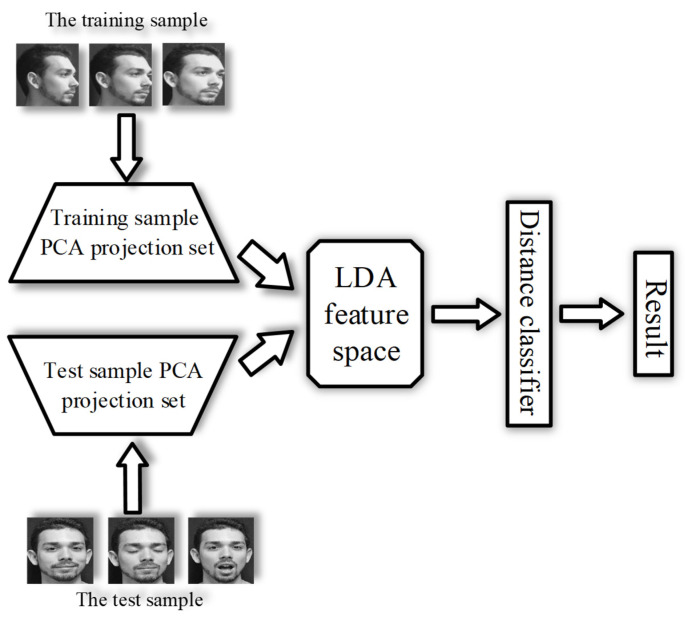
Face recognition process based on the PCA-LDA algorithm.

Figure 5 shows the PCA-LDA algorithm’s recognition process. First, the PCA is applied to project the high-dimensional space into the low-dimensional feature space. Second, the LDA and parameter optimization methods are proposed to obtain the fused features based on the low-dimensional feature space. Third, the training and test samples are projected on the fused feature space. Last, the classification of a test sample is carried out based on the Euclidean distance.

### 3.5. Analysis of Experimental Results

#### 3.5.1. Experimental Analysis of Face Image Fusion

Table 5 shows the time taken for image fusion. The results show that the fusion speed of FTSGAN model is the fastest on graphics card and CPU, which are equal to 0.02 and 0.16 s/p, respectively. We optimized the model’s structure and reduced the number of layers, relying more on the loss function to achieve the model generation quality and obtain fast fusion performance. (The CNN [30] is a Siamese network in which the weights of the two branches are constrained to the same and each branch consists of three convolutional layers and one max-pooling layer).

**Figure 6:**
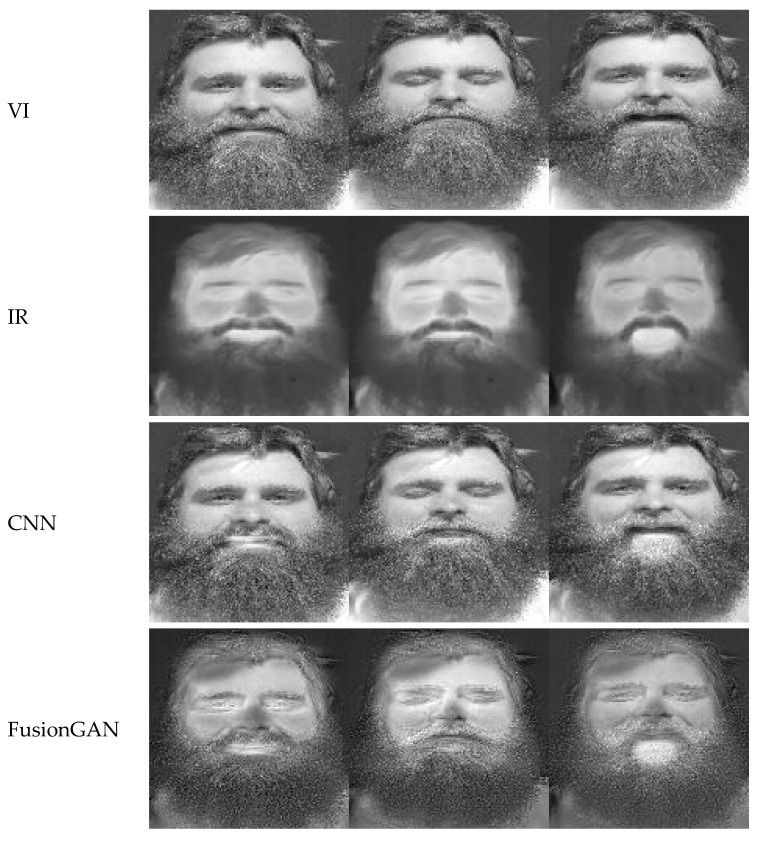

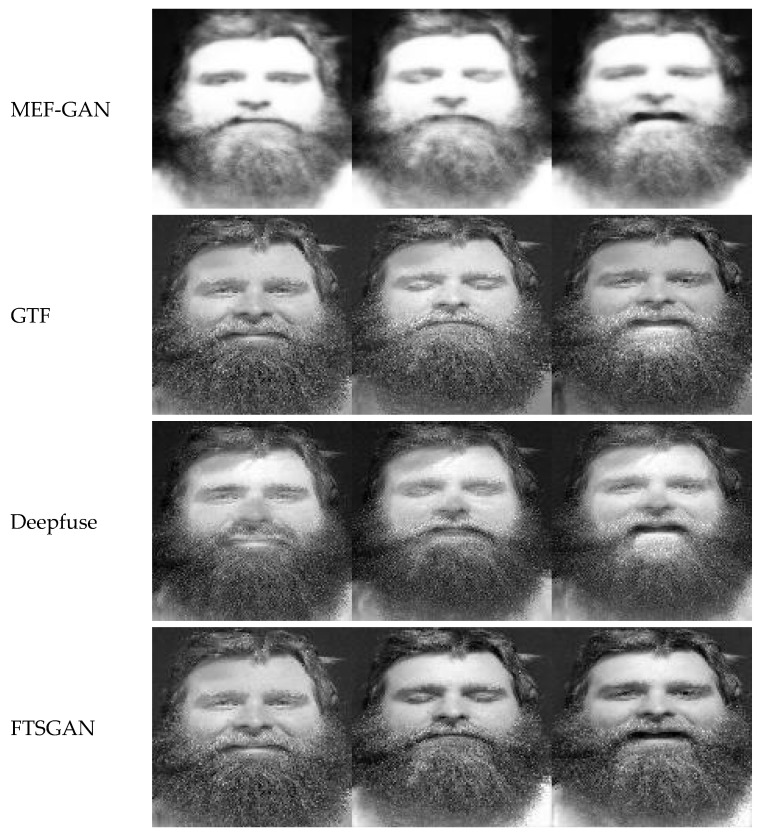
Performance with different models before and after the fusion of VI and IR. VI—visible image; IR—infrared image.

As Figure 6 shows, the images generated by CNN, GTF, and FTSGAN models are clearer with distinct gradients, and richer in texture compared to the original images. The images obtained using the FTSGAN are less noisy and have clearer light and dark gradients. The images generated by the FusionGAN model have textures that are more inclined towards the infrared images, and the details of visible images are significantly lost. The images generated by the MEF-GAN model are blurred and show a serious loss of texture in visible and infrared images. The images generated by the fusion of Deepfuse model are somewhat blurred compared with the CNN, GTF, and FTSGAN models. These images suffer serious gradient loss and are very noisy. Therefore, our FTSGAN model performs the best in fusing visible and infrared images.

We adopted the image quality metrics such as entropy [34], standard deviation (*SD*) [35], mean gradient (*MG*), spatial frequency (*SF*), peak signal-to-noise ratio (*PSNR*) [36], and gradient-based fusion performance (*QG/QABF*) [15] to examine the quality of multiple image fusion models. These metrics are defined as follows:

Entropy (*EN*): An objective evaluation metric that measures how much information an image contains. It is calculated as:

(6) $$EN=-\overset{L-1}{\underset{i=0}{\sum }}p_{i}log_{2}p_{i}$$

where $p_{i}$ denotes the normalized histogram of the corresponding gray level in the fused image, and the number of all the gray levels is set as L. A higher entropy means a higher amount of information and a better image quality.

Standard deviation (*SD*): It indicates the shift of the pixel value of an image relative to the average pixel value of the image. The larger the *SD*, the better the visual quality of the image. It is defined as follows:

(7) $$SD=\sqrt{\frac{1}{MN}\overset{M}{\underset{i=1}{\sum }}\overset{N}{\underset{j=1}{\sum }}{\left(x_{i,j}-\mu \right)}^{2}}$$

where μ denotes the average pixel value of the image.

Mean gradient (*MG*): It reflects the texture information of the image and is calculated as:

(8) $$MG=\frac{1}{\left(M-1\right)\left(N-1\right)}\overset{M}{\underset{i=2}{\sum }}\overset{N}{\underset{j=2}{\sum }}\sqrt{\frac{{\left(x_{i,j}-x_{i-1,j}\right)}^{2}+{\left(x_{i,j}-x_{i,j-1}\right)}^{2}}{2}}$$

A larger *MG* signifies that a higher gradient information is contained in the image, the change of the pixel values is higher, and the image is sharper.

Spatial frequency (*SF*): It represents the change rate of the image grayscale, reflecting the image details and textures based on the image gradient. The *SF* function is defined by the spatial row frequency (*RF*) and column frequency (*CF*). It is shown as follows:

(9) $$SF=\sqrt{RF^{2}+CF^{2}}$$

where $RF=\sqrt{\frac{1}{MN}\sum_{i=1}^{M}\sum_{j=2}^{N}{\left(x_{i,j}-x_{i,j-1}\right)}^{2}}$ and $CF=\sqrt{\frac{1}{MN}\sum_{i=2}^{M}\sum_{j=1}^{N}{\left(x_{i,j}-x_{i-1,j}\right)}^{2}}$. The larger the value of *SF*, the richer the edge texture information contained in the image, and the better its quality.

Peak signal-to-noise Ratio (*PSNR*): It is generally applied for standard calculations between maximum signal and background noise. It is calculated as:

(10) $$MSE=\frac{1}{mn}\overset{m-1}{\underset{i=0}{\sum }}\overset{n-1}{\underset{j=0}{\sum }}||I\left(i,j\right)-K\left(i,j\right)|{|}^{2}$$

(11) $$PSNR=10\cdot \log_{10}\left(\frac{MAX_{I}^{2}}{MSE}\right)$$

where *MSE* is the mean square error and $MAX_{I}^{2}$ denotes the square of the largest value of the image point color. Therefore, a larger *PSNR* value means less distortion and better image quality.

Gradient-based fusion performance (*QG/QABF*): This metric reflects the quality of the visual information obtained from the input image fusion. It is defined as follows:

(12) $$Q\left(a,b,f\right)=\frac{1}{\left|W\right|}\underset{\omega \in W}{\sum }\left(\lambda \left(\omega \right)Q_{0}\left(a,f|\omega \right)+\left(1-\lambda \left(\omega \right)\right)Q_{0}\left(b,f|\omega \right)\right)\;$$

where *a* and *b* represent the two images in different bands, respectively, and *f* represents the fused image. A higher value of *QABF* indicates a better quality of the fused image.

**Figure 7:**
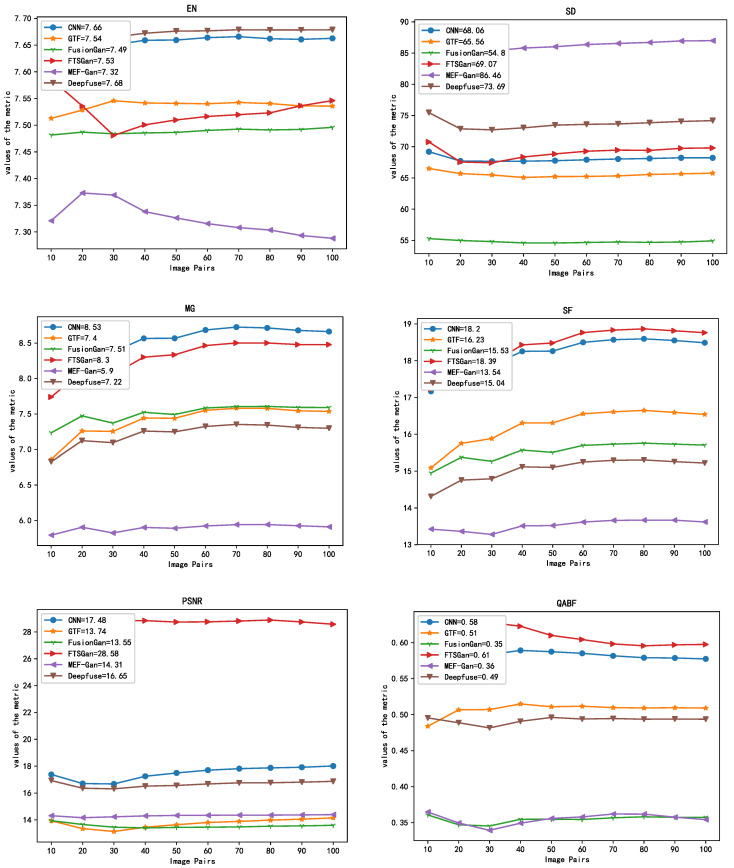
Evaluation of six fusion methods for infrared and visible image fusion. The metrics corresponding to each method are given in the legends.

Figure 7 shows that the proposed FTSGAN model has the maximum *PSNR*, *QABF* and *SF* values. The model significantly outperforms the other models, especially on the *PSNR* metric. The proposed method performs second-best on the *MG* metric, while the *EN* and *SD* metrics are in the middle of the range. These metrics demonstrate that our model can (1) preserve the maximum information of images for fusion and improve the quality of the fused image to a certain extent, (2) enhance the information of the image’s edge gradient, and (3) increase the information content of the image.

**Figure 8:**
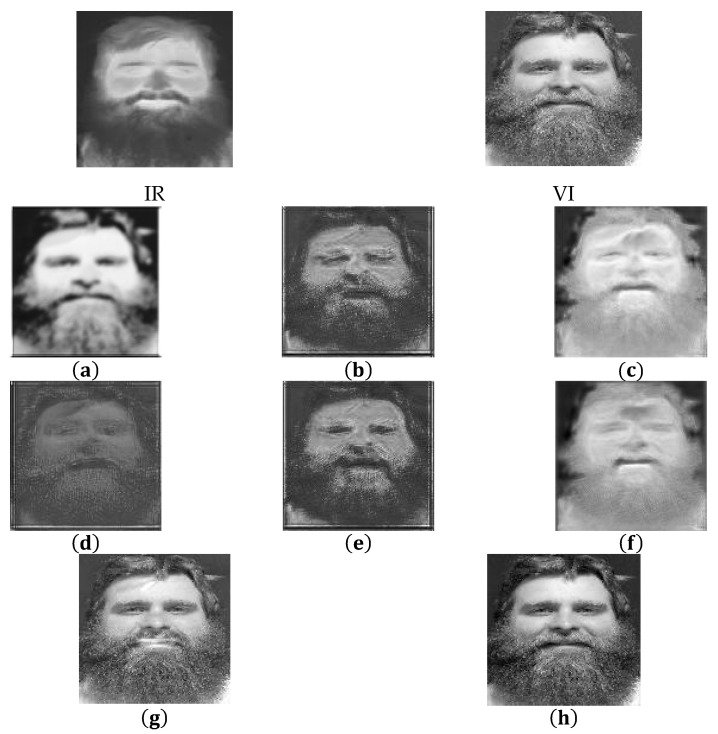
The result of changing the loss function of $L_{G}$ on image fusion. (**a**) $L_{G}^{a}$, (**b**) $L_{G}^{a}+SSIM$, (**c**) $L_{G}^{a}+TV$, (**d**) $L_{G}^{a}+$ *F*, (**e**) $L_{G}^{a}+$ *SSIM* + *F*, (**f**) $L_{G}^{a}+$ *TV* + *F*, (**g**) $\;L_{G}^{a}+$ *TV* + *SSIM*, (**h**) $\;L_{G}^{a}+F+TV+SSIM$.

By means of the original loss function of Gan, the generated image edge is blurred. In the case of antagonistic loss, the fusion result cannot show more and clearer texture details in the visible image, as shown in Figure 8a. In Figure 8b, we added *SSIM* to the loss function. Then, we found that the depth of the image was enhanced, but the contour of the image was also blurred. In Figure 8c, we added *TV* to the loss function. The edge of the image became clear. However, the depth of the image became shallow, which reduced the visibility of the image. In Figure 8d, the *F* norm was used to change the depth of the image, but the edges were still blurred. In Figure 8e, the combination of *SSIM* and *F* was applied to increase the visibility and depth of the image, but the contour problem of the edge was still not solved. In Figure 8f, we added *TV* and *F* to the loss function. The depth the image was enhanced and the edge of the image was clear, but it was still not enough. In Figure 8g, we added *TV* and *SSIM* to the loss function. The *TV* and *SSIM* could combine the characteristics of infrared and visible images, and the depth and edge information of the image was clear. In Figure 8h, we added *F*, *TV*, and *SSIM* to the loss function. The *F*, *TV*, and *SSIM* made the quality of the image further improved. We achieved good results in the improvement of the loss function.

#### 3.5.2. Face Recognition Effect Analysis

(1) Selection of training face dataset

Face recognition accuracy is low for both the front and side face images. The reasons are as follows: (1) The light intensity in a few face images is insufficient. (2) The gray level of the image does not obey the normal distribution. (3) The contrast is not apparent. When we experiment with the side face images, the features of the front face images cannot be expressed effectively, which decreases the recognition accuracy of the face images. In the PCA-LDA algorithm, we avoided applying the side face images as the training dataset. Besides, to consider the reflection of light by the glasses, we divided the dataset into a dataset with eyeglasses and one without eyeglasses. We divided every set evenly so that each fold contains two pictures.

(2) Experimental results

**Figure 9:**
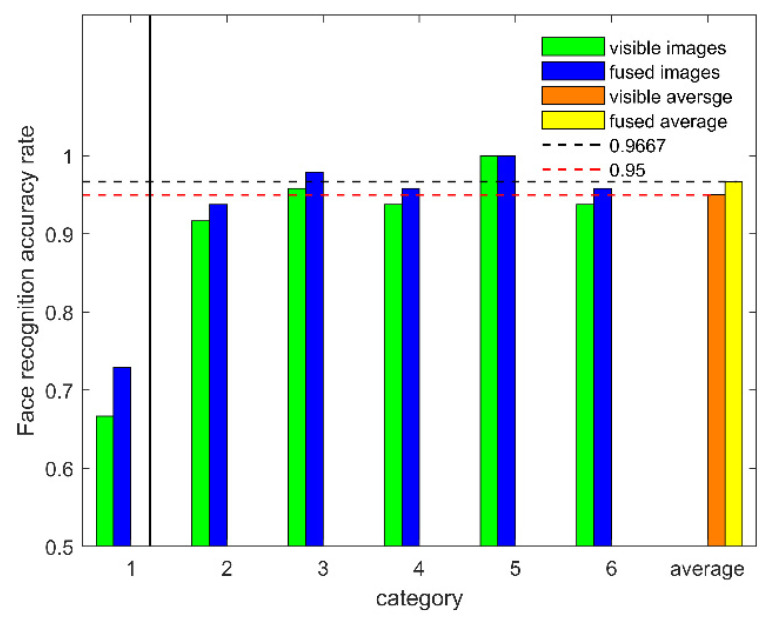
Face recognition accuracy.

A quantitative comparison experiment on the accuracy of visible images’ face recognition and the accuracy of fused images’ face recognition was carried out with a dataset containing face images with eyeglasses. Table 6 and Figure 9 show the face recognition accuracy and the CPU time required for recognition, respectively. In 6-fold, 1-fold was eliminated based on Section 3.5.2. (1) of this paper. When experimenting with 5-fold, the average accuracy increase was equal to 1.7%. The above data show that our FTSGAN enhanced the quality of images under the condition of face images with eyeglasses.

**Figure 10:**
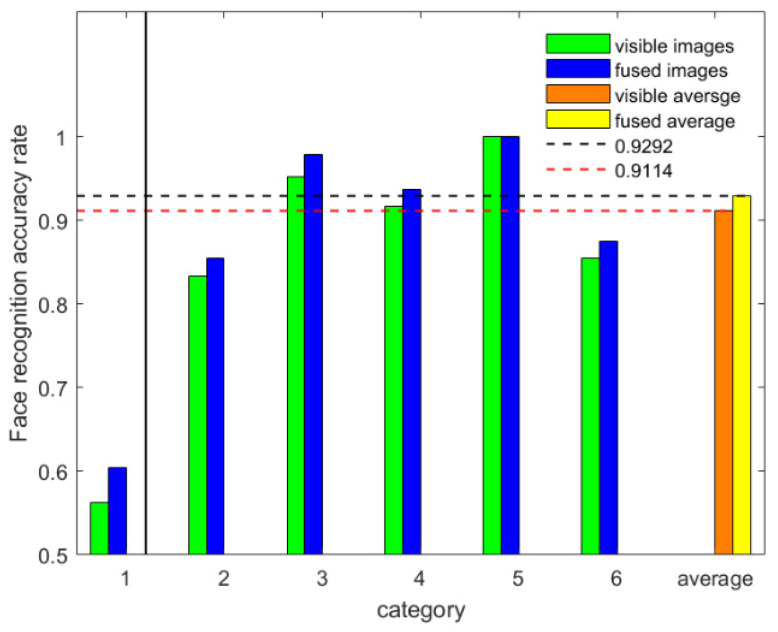
Face recognition accuracy.

The face recognition accuracy improved under the condition of without wearing the eyeglasses, as presented in Table 7 and Figure 10. Table 7 shows the recognition time taken by the CPU. In 6-fold, the 1-fold was eliminated as described in Section 3.5.2. (1) of this paper. According to the data given in Table 7, we could observe an average accuracy increase of 1.8%. The increase indicated that regardless of whether the subjects wear eyeglasses or not, the fused image quality was not affected.

**Figure 11:**
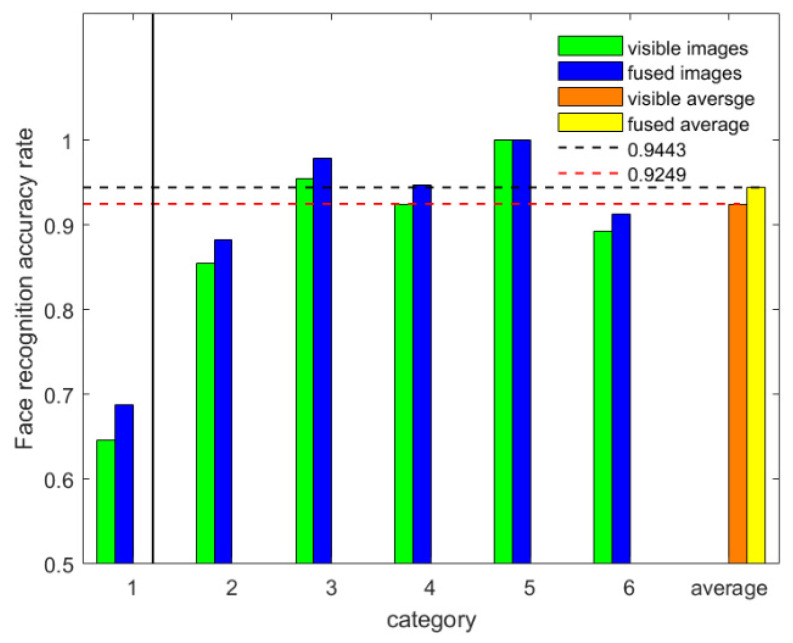
Face recognition accuracy.

Table 8 and Figure 11 show that the face recognition accuracy of the fused images is higher than that of the visible images under the condition of all images being used in the experiment. In 6-fold, the 1-fold is eliminated as described in Section 3.5.2. (1) of this paper. The average accuracy increased is 1.9%. The recognition time taken by the CPU is shown in Table 8. The recognition accuracy of fused images was always higher than that of visible images. This indicates that our FTSGAN has excellent experimental results and provides solid theoretical foundation for further practical applications.

The data given in the above three tables and [s show that the proposed FTSGAN successfully fuses visible and infrared images. The face recognition accuracy of the fused images is 1.9% higher than that of the visible images.

## 4. Conclusions

Compared with other fusion models, the proposed FTSGAN obtained remarkable image fusion performance over six evaluation metrics. The FTSGAN could improve the image quality required for face recognition. We used the PCA-LDA face recognition algorithm to verify this result. The face recognition performance after fusion improved by 1.9% compared to that before fusion, and the final face recognition rate was 94.4%. In addition, the FTSGAN model outperformed all the other exiting models.

The experiments proved that face images were best recognized using the frontal face, and the information of side faces was prone to generate false results. The use of side face photos should be avoided when applying this algorithm. Additional information can also be used to complement face recognition in future work, such as compensation using human eye iris information and face temperature information. Due to the lightweight feature of the fusion model, we will implement it on handheld devices or embedded operating systems in the future.

## Figures and Tables

**Table 1:** Information about the hardware devices used for our experiments.

**Equipment**	Graphics Board	Central Processing Unit
**Equipment parameters**	RTX2060 (notebook)-6GB	INTEL Core-I7-9750H

**Table 2:** Information on the face experiment data needed for our experiments.

Data Source	Properties							
Tufts University face data	**Sex**		**Shelter**		**Visible Light**	**Infrared Light**	**Aligned or Not**	**Pixel**
	male	female	glass	no_glass	480(40 × 12)	480(40 × 12)	Aligned	128 × 128
	20	20	20 pairs	20 pairs				

**Table 3:** Overall experimental procedure.

Experimental Steps	Experimental Projects
**1**	- Preprocessing of the Tufts University dataset.
**2**	- The FTSGAN is trained using the processed data. The trained model generates the face fusion image dataset.
**3**	- The face database is added to the designed face recognition algorithm for identity verification.
**4**	- Summary of experimental performance.

**Table 4:** FTSGan model training process.

Training Process of Visible and Infrared Fusion
Parameter descriptions: $L_{MIN}$: The minimum G loss function after training $L_{MIN}$: The minimum D loss function after training D, G: Discriminator and Generator $L_{D},\;\;L_{G}$: Loss functions of Discriminator and Generator
$\mathrm{Initialize}\;\theta_{D}$ $\;\mathrm{for}\;D\mathrm{and}\;{\;\theta }_{G}\;\mathrm{for}\;G$
In each training iteration:
-Train Discriminator D
-N paired infrared and visible data pairs
-Train the discriminator to obtain multiple pairs of data
-$\mathrm{Update}\;\mathrm{discriminator}\;\mathrm{parameters}\;\theta_{D}$$\;\mathrm{using}\;\mathrm{the}\;\mathrm{AdamxOptimizer}\;\mathrm{to}\;\mathrm{minimize}\;L_{D}$$\;\mathrm{in}\;\mathrm{Equation}\;(5).\;\mathrm{If}\;L_{D}<L_{MIN}$ in the next five epochs, repeat Train Generator D.
-Train Generator G.
-Concatenate stacks-N pairs of paired infrared and visible data
-Train the generator to obtain multiple pairs of fused images
-$\mathrm{Update}\;\mathrm{generator}\;\mathrm{parameters}\;\theta_{G}$$\;\mathrm{using}\;\mathrm{the}\;\mathrm{AdamxOptimizer}\;\mathrm{to}\;\mathrm{minimize}\;L_{G}$$\;\mathrm{in}\;\mathrm{Equation}\;(2).\;\mathrm{If}\;L_{G}<(L_{MIN}\;\pm \;5)$ in the next five epochs, repeat Train Generator G.
Every 20 epochs: -Evaluate the generator fusion model for generating image quality.

**Table 5:** Time of image fusion. The fusion speeds of the six fusion models were tested separately on graphics card and CPU. The graphics card and CPU were tested using the parameters given in Table 1. (‘s/p’ is ‘seconds/pair-image’).

Species		CNN [30]	GTF [31]	MEF-Gan [32]	FusionGan [13]	Deepfuse [33]	FTSGan
**Average fusion time**	**Graphics card**	0.14 s/p	0.12 s/p	0.08 s/p	0.04 s/p	0.03 s/p	0.02 s/p
	**CPU**	7.2 s/p	4.2 s/p	4.3 s/p	0.46 s/p	0.34 s/p	0.16 s/p

**Table 6:** Comparison of the face recognition accuracy.

K-Fold	Fusion	Visible	Ft	Vt
6-fold	72.92%	66.67%	0.98 s	0.75 s
	93.75%	91.67%	0.77 s	0.55 s
	97.92%	95.83%	0.70 s	0.61 s
	95.83%	93.75%	0.82 s	0.69 s
	100%	100%	0.46 s	0.45 s
	95.83%	93.75%	0.76 s	0.60 s
average	96.67%	95%	0.70 s	0.58 s

Ft: Time required for face recognition in fused images; Vt: Time required for face recognition in visible images.

**Table 7:** Comparison of the face recognition accuracy.

K-Fold	Fusion	Visible	Ft	Vt
6-fold	60.42%	56.25%	0.63 s	0.59 s
	85.42%	83.33%	0.55 s	0.49 s
	97.92%	95.26%	0.62 s	0.50 s
	93.75%	91.67%	0.66 s	0.49 s
	100%	100%	0.35 s	0.33 s
	87.5%	85.42%	0.72 s	0.67 s
average	92.92%	91.14%	0.58 s	0.50 s

**Table 8:** Comparison of the face recognition accuracy.

K-Fold	Fusion	Visible	Ft	Vt
6-fold	68.75%	64.58%	0.86 s	0.55 s
	88.21%	85.42%	0.80 s	0.60 s
	97.92%	95.41%	0.88 s	0.63 s
	94.72%	92.43%	0.72 s	0.55 s
	100%	100%	0.79 s	0.66 s
	91.32%	89.21%	0.76 s	0.60 s
average	94.43%	92.49%	0.79 s	0.61 s

## Data Availability

Our study does not report any data.
